# Assisted HIV partner notification services in resource‐limited settings: experiences and achievements from Cameroon

**DOI:** 10.1002/jia2.25310

**Published:** 2019-07-19

**Authors:** Pius M Tih, Francois Temgbait Chimoun, Eveline Mboh Khan, Emmanuel Nshom, Winifred Nambu, Ray Shields, Beatrice M Wamuti, Matthew R Golden, Thomas Welty

**Affiliations:** ^1^ AIDS Care and Prevention Program Cameroon Baptist Convention Health Services Bamenda Cameroon; ^2^ Department of Global Health University of Washington Seattle WA USA; ^3^ Department of Epidemiology University of Washington Seattle WA USA; ^4^ Public Health Seattle & King County HIV/STD Program Seattle WA USA

**Keywords:** partner notification, HIV testing, HIV case finding, linkage to care, HIV prevention, Cameroon

## Abstract

**Introduction:**

In 2007, the Cameroon Baptist Convention Health Services (CBCHS) initiated an assisted partner notification services (aPNS) public health programme to increase HIV case identification and reduce HIV incidence in the most affected regions of Cameroon. We describe large‐scale implementation of aPNS and overall programmatic achievements in a resource‐limited setting through 2015.

**Methods:**

CBCHS trained health advisors (HAs) from 16 CBCHS facilities and 22 non‐CBCHS facilities to integrate aPNS into their existing jobs in five of the ten Cameroon regions. HAs recorded basic demographic, clinical and risk factor information from consenting index persons (IPs) and similar information about their sexual partners’/contact persons (CPs) on interview records and aPNS registers. These data were entered into an Epi‐Info database. HAs provided pre‐test counselling to CPs and offered them HIV testing in their home or other location. HAs educated IPs and CPs on HIV prevention and risk reduction, and referred IPs and HIV positive CPs to HIV care and treatment centres. Starting in 2014, HAs re‐interviewed IPs 30 days after their initial aPNS interview to ascertain instances of social harms following partner notification. Continuous predictor and outcome variables were summarized using median and interquartile range, while categorical variables were summarized using percentages from 2007 to 2015.

**Results:**

A total of 18,730 IPs (71% women) received aPNS over nine years. IPs identified 21,057 CPs (67% men) (mean CP/IP 1.12), of whom 12,867 (61.1%) were notified of their exposure to HIV. A total of 9202 (71.5% of notified CPs) tested for HIV, 4764 (51.8%) of whom tested HIV positive (number of IPs needed to interview = 3.9); 3112 (65.3%) HIV‐positive partners were referred to HIV care and treatment centres. Of the 976 IPs receiving aPNS in 2014 to 2015, for whom follow‐up data were available, 11 (1.1%) reported physical intimate partner violence from CPs. Thus, 44.3% of 1224 CPs were notified through provider referral. Of the 784 CPs who tested for HIV, 157 were newly diagnosed and the overall HIV prevalence was 41.6% (326/784).

**Conclusions:**

aPNS is feasible, can be brought to scale, yields a high level of case identification, and is infrequently associated with social harms and intimate partner violence.

## 1 Introduction

Increased HIV testing of high‐risk groups and linkage of those who are HIV positive to antiretroviral treatment (ART) is needed to achieve the 95‐95‐95 objectives by 2030 (95% of people living with HIV (PLHIV) diagnosed; 95% of diagnosed people on ART; and 95% of all people receiving ART having viral suppression) 1. The Cameroon Population‐based HIV Impact Assessment, a 2017 household‐based survey, estimated that 500,000 PLHIV aged 15 to 64 years residing in Cameroon, which had an HIV adult prevalence of 3.7% and 53% of PLHIV, were unaware of their status 2. HIV transmission in Cameroon primarily occurs through heterosexual sex, and women suffer a disproportionate burden of infection 3. Assisted partner notification services (aPNS), previously called contact tracing, have been implemented as a public health intervention to identify individuals most at risk of HIV infection, screen those who consent and link those who are HIV positive to treatment 4, 5.

Contact tracing has been integrated into public health efforts to control sexually transmitted infections, including HIV, in some parts of the United States and Europe for a long time 6, 7. However, it has not traditionally been part of HIV prevention and care in low‐ and middle‐income nations. Instead, most clinicians and public health programmes have counselled patients to notify sexual partners/contact persons (CPs) by themselves – a form of passive referral – and the success of this approach has not been consistently measured 8. Based on a contact tracing model used in the North Carolina Department of Health, which was derived from tools developed by the United States Centers for Disease Control and Prevention (CDC) in the 1990s, the Cameroon Baptist Convention Health Services (CBCHS) initiated aPNS in 2007. To the best of our knowledge, this was the first large‐scale implementation of its kind in Cameroon and in SSA. In December 2016, the World Health Organization (WHO) released new guidelines on HIV self‐testing and partner notification 9 based on evidence from clinical trials and observational studies, mostly from SSA countries such as Kenya, Malawi, Mozambique and Cameroon 10, 11, 12, 13. aPNS is now acknowledged as an effective strategy in the HIV epidemic control 4, 14.

In this article, we describe the initiation, implementation and programmatic achievements of the CBCHS aPNS programme from its inception in 2007 through 2015.

## 2 Methods

### 2.1 Programme initiation and implementation

CBCHS started aPNS in 2007 as a public health intervention without support from national and international partners. At that time, WHO guidelines did not recommend the routine provision of the strategy. The programme relied entirely on CBCHS frontline health advisors (HAs), who were trained using training materials adapted from CDC contact tracing tools. The first cohort of 15 HAs trained for the programme included HIV educators, laboratory technicians, nurses, social workers and chaplains, who provided aPNS in addition to their usual jobs.

aPNS began in one CBCHS health facility located in the Northwest Region of Cameroon and expanded to 15 other CBCHS facilities in five of the ten regions by the end of 2015 through technical assistance and financial support received from the Elizabeth Glazer Pediatric AIDS Foundation, Centers for AIDS Research at the University of Washington and the University of North Carolina, after the programme documented early promising results.

In 2013, aPNS were integrated into other HIV and reproductive health services in 22 non‐CBCHS facilities selected as pilot sites for the prevention of mother to child transmission (PMTCT) programme Option B+ strategy before Cameroon's national scale‐up of Option B+.

### 2.2 Programme procedures

HAs were trained in the effective use and interpretation of the HIV rapid diagnostic test (Determine HIV 1 and 2), data collection, and the management of social harms, including intimate partner violence (IPV) and partnership dissolution occurring after index persons (IPs) received aPNS. If the HIV test performed by the HA was positive, the HA referred the CP to a health facility for confirmatory testing. HAs counselled HIV‐positive clients on how to reduce their risk of HIV transmission and HIV negative CPs on preventing HIV acquisition. HAs followed up participants, and engaged supervisors, pastors and social workers when they suspected or identified social harms, with the intention of minimizing the risk of physical, sexual and psychological harm. At the end of the training, HAs agreed to ensure the confidentiality of clients, both verbally and by signing a non‐disclosure agreement (see Appendix S1).

In providing aPNS, CPs were the primary target of HAs, although in some instances, the HAs proposed and tested other household contacts including children after obtaining their verbal consent. Data from this sub‐population were not systematically collected and are therefore not included in this analysis.

Whenever possible and after seeking verbal consent, HAs started aPNS on the same day that the IP was diagnosed HIV positive. HAs offered IPs three options to notify their sex partners 9:

#### 2.2.1 A provider or active referral

The HA with consent from the IP takes the responsibility to confidentially contact CP(s) identified by the IP and to notify CP(s) of their exposure to HIV.

#### 2.2.2 Contract, conditional, hybrid or negotiated referral

The HA and the IP make a contract, whereby the IP agrees to disclose his/her HIV status to CP(s) and encourages the CPs to test within a specified period, usually within two weeks. If the CP(s) do not test during that period, the provider contacts the CP(s) directly.

#### 2.2.3 Patient referral

The HA counsels the IP, often using role plays, to disclose their HIV status to CP(s) and the IP then encourages the partners to come to a facility for testing.

HAs and IPs agreed together on the best approach to inform CPs. However, the programme promoted provider referral because direct communication between HAs and CPs yielded more positive results compared to other options. Under provider referral, HAs notified CPs of their potential exposure to HIV either in person or by telephone. HAs pre‐test counselled CPs and offered them HIV testing either in the health facility, their home or they were referred to another health facility for testing. HAs followed up through regular phone calls to confirm that the CP(s) had tested for HIV and recorded the test results in an aPNS register and kept it in a locked office. Finally, HAs provided education on HIV prevention and referred IPs and HIV positive CPs to HIV care and treatment services. HAs documented referral of HIV positive CPs, but did not systematically ensure enrolment, retention in care and adherence to ART due to lack of resources and training.

### 2.3 Data collection, monitoring and evaluation

HAs obtained verbal consent prior to data collection by explaining the purpose and content of aPNS to both IPs and CPs, and reiterated that they were free to either accept or decline aPNS without affecting access to other services at the facility. Information collected during face‐to‐face or phone interviews was recorded on interview forms and stored in a secure place at a health facility or in the community. Although interview forms varied throughout the evaluation period to capture critical information or to streamline the process, all records included information on basic demographics, sexual risk behaviour, HIV testing history, sexual partner details and aPNS outcomes. These data were used to guide counselling, identify partners requiring testing and offer monitoring and evaluation data.

HAs transferred data from interview forms into aPNS registers secured within each health facility. Monthly facility reports of aggregate registry data were then sent to the central CBCHS aPNS programme office where they were merged into the overall aPNS workload reports. HAs also sent completed interview forms to the central aPNS programme office periodically for entry into an Epi Info database. Incomplete interview forms were retained in participating facilities until follow‐up data were recorded although many were never forwarded to the central aPNS programme office for final data entry. Although the Epi Info database lacks complete workload data, it provides more detailed analyses for programme monitoring and evaluation. The computer databases were password protected with access limited to programme staff who have signed the confidentiality form.

In 2014 to 2015, the CBCHS programme received funding to allow HAs to actively follow‐up on IPs 30 days after their initial interview, and assess social harms and IPV associated with aPNS. At these follow‐up interviews, the HAs recorded IPs responses on the same interview form used at the initial visit. The results of both the initial and follow‐up interviews were then sent to the central office for entry into the Epi Info database.

### 2.4 Statistical analysis

We report two analyses. First, using aggregate data from registries between 2007 and 2015, we report total programme outcomes, including the number of IPs receiving aPNS, the number and percentage of CPs reported, notified, tested, and referred for care and treatment. For this analysis, we also report the number of IPs needed to interview (NNTI) to identify one CP with HIV, that is, number of IPs receiving aPNS divided by the number of CPs testing HIV positive. Second, using data from interview forms entered in an Epi‐Info database from 2014 to 2015, we reported the characteristics of IPs and CPs, methods used to notify CPs and the occurrence of social harms, including those described on follow‐up. Continuous predictor and outcome variables were summarized using the median and interquartile range (IQR), while categorical variables were summarized using percentages. The analysis was conducted using Stata 14.0 (StataCorp, College Station, TX, USA).

### 2.5 Ethical considerations

Ethical approval was sought and obtained from the CBCHS Institutional Review Board (IRB) before the programme inception. The IRB deemed aPNS to be a public health intervention, which should be monitored and evaluated, and did not consider it research.

## 3 Results

### 3.1 Overall programme aPNS achievements

**Figure 1:**
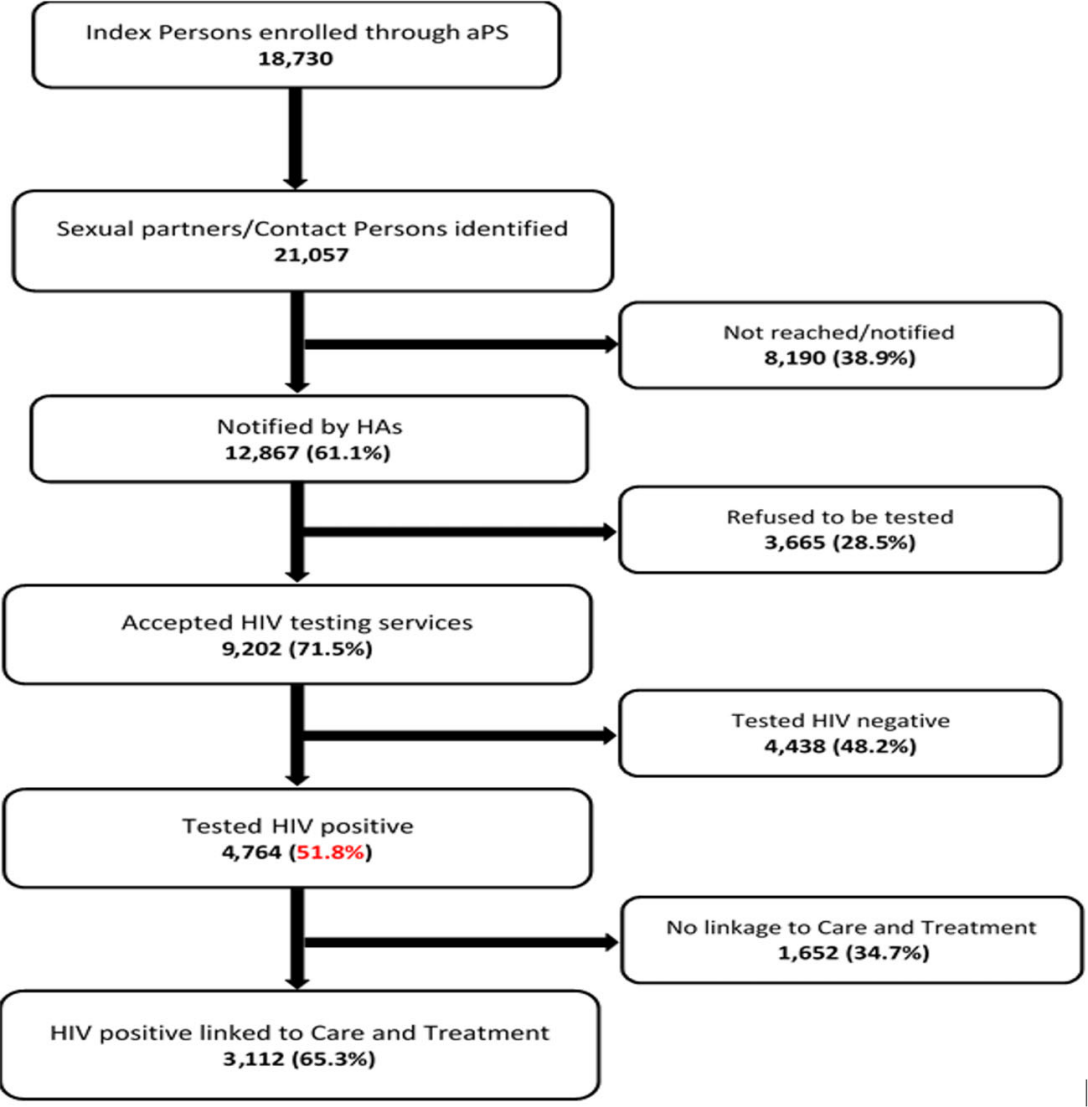
**Assisted partner notification services flow chart of aggregate data from 2007 to 2015 (percentages throughout use denominators from the box in the preceding step).**

Figure 1 shows the flow chart of aPNS in Cameroon from inception in 2007 through December 2015. Overall, 18,730 IPs consenting to receive aPNS identified 21,057 CPs to notify and test. Of these CPs, 12,867 (61%) learned of their exposure to HIV, 9202 (44% of all CPs and 71% of notified CPs) tested for HIV and 4764 (23% of all CPs, 37% of notified CPs and 52% of tested CPs) tested HIV positive. The NNTI to identify one CP with HIV infection was 3.9.

Table 1 presents the annual programme data from 2007 to 2015. The majority of the IPs were women (68%) while the majority of CPs were men (67%). After an initial period of scale‐up from 2007 to 2009, the number of IPs receiving aPNS varied from 2061 in 2011 to 3030 in 2014. The annual contact/index was stable throughout the programme, ranging from 1.1 to 1.3. However, the programme's success identifying CPs was variable, peaking in 2010 when 969 CPs tested HIV positive and the NNTI varied from 2.7 in 2010 to 5.5 in 2015.

**Table 1:** Number of index cases and contact persons (CPs) receiving assisted partner notification services per year, and programme outcomes, 2007 to 2015, derived from aggregate data

	2007	2008	2009	2010	2011	2012	2013	2014	2015	Total
Index persons (IPs)	227	1610	2174	2587	2061	2409	2439	3030	2193	18,730
IPs male (%)^a^	–	464 (29)	713 (33)	873 (34)	679 (33)	775 (32)	806 (33)	766 (25)	790 (36)	5866 (31.7)
IPs female (%)^a^	–	1146 (71)	1461 (67)	1713 (66)	1382 (67)	1634 (68)	1633 (67)	2264 (75)	1403 (64)	12,636 (68.3)
CPs	278	1701	2384	2812	2476	3041	2710	3283	2372	21,057
CPs male (%)^a^	–	1182 (69)	1616 (68)	1899 (67)	1593 (64)	2038 (67)	1773 (65)	2404 (73)	1466 (62)	13,971 (67.2)
CPs female (%)^a^	–	519 (31)	768 (32)	913 (33)	883 (36)	1003 (33)	937 (35)	879 (27)	906 (38)	6808 (32.8)
Number of named CP per IP	1.2	1.1	1.1	1.1	1.2	1.3	1.1	1.1	1.1	1.1
CPs notified	167	1309	1742	2184	1416	1627	1336	1981	1105	12,867
CPs tested	110	1004	1477	1681	808	1139	863	1339	781	9202
CPs HIV positive	55	557	688	969	446	588	470	592	399	4764
CPs HIV positive referred to care and treatment	0	37	90	633	302	587	470	591	399	3112
NNTI^b^	4.1	2.9	3.2	2.7	4.6	4.1	5.2	5.1	5.5	3.9

^a^Sex distribution of IPs and CPs not available for 2007; ^b^NNTI: number of IPs needed to interview to identify one CP with HIV.

### 3.2 Notification, HIV testing, and referral to care and treatment of CPs

**Figure 2:**
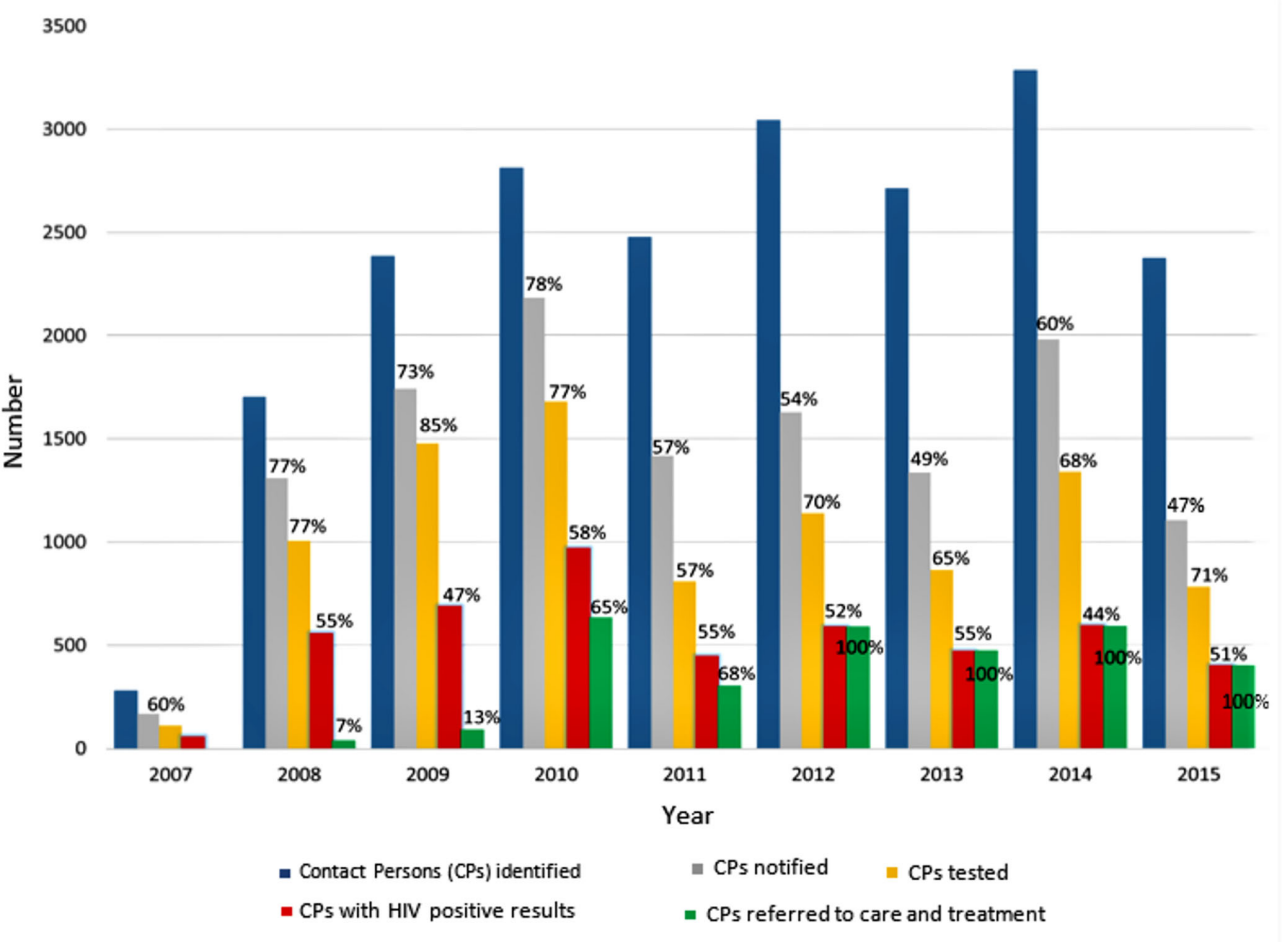
**Number of contact persons (CPs) per year and number of CPs achieving each assisted partner notification services, 2007 to 2015. (Percentages above bars are the percentage of CPs with each outcome.)**

Figure 2 presents data on trends in the number of CPs identified, tested, and diagnosed with HIV from 2007 to 2015. A total of 12,867 CPs (61.1%) were notified. After pre‐counselling sessions, 9202 (71.5%) of them accepted and received HIV testing services (HTS) (Table 1). From 2011 to 2015, the average percentage of CPs notified per year was 53.4% with the lowest level of 47% in 2015.

The overall HIV prevalence was 51.7% among the 9202 CPs who were tested. The highest HIV prevalence was recorded in 2010 with 58% (Figure 2). Overall, 66% of HIV positive CPs were referred to HIV care and treatment. Referral increased each year between 2007 and 2012, and from 2012 onward, all HIV positive CPs were referred to care (Figure 2).

### 3.3 Demographic characteristics of IPs and CPs: sub‐set data from 2014 to 2015

Information on interview forms from 1261 IPs and 1357 CPs who were followed up was entered into the Epi Info database in 2014 to 2015 and analysed. Almost all CPs (95.6%) identified by IPs were notified of their exposure. The socio‐demographic characteristics of IPs and CPs are presented in Table 2. The median age for both groups was 36 years (IQR: 30 to 43). The majority of IPs (63.8%) were female, married (52%) and 70.1% were seen in a rural facility. On follow‐up, most IPs had enrolled in an HIV care clinic with 72.6% having a CD4 count and 59.7% receiving ART. During 2014 to 2015, 5223 IPs received aPNS, but only 1261 had follow‐up data entered into the Epi Info database.

**Table 2:** Demographic and HIV testing characteristics for index persons (IPs) and contact persons (CPs)

	IPs N = 1261	
	N/median	%
Demographic characteristics IPs		
Gender		
Female	805	63.8
Male	456	36.2
Marital status		
Single	317	25.1
Married monogamous	603	47.9
Married polygamous	53	4.2
Divorced	124	9.8
Widow/widower	109	8.6
Missing	54	4.3
Sites		
Rural	884	70.1
Urban	376	29.8
Other (not specified)	1	0.1
HIV care outcomes IPs		
CD4 count done at diagnosing site		
Yes	916	72.6
No	32	2.5
Missing	313	24.8
IPs on anti‐retroviral therapy		
Yes	753	59.7
No	185	14.7
Missing	323	25.6

	CPs N = 1357	
	N	%
Demographic characteristics of CPs		
Gender		
Male	832	61.3
Female	515	38.0
Missing	10	0.7
Relationship with IPs		
Wife/husband	773	57.0
Girlfriend/boyfriend	549	40.5
Other	11	0.8
Missing	24	1.8

### 3.4 HIV care outcomes CPs notified: sub‐set data from 2014 to 2015

Of the 1357 identified CPs, 1224 (90.2%) were traced for notification, of whom 1170 (95.6%) were successfully notified (Table 3). IPs elected to notify 43.7% via patient referral and elected to use provider referral for 44.3%. Most partners were notified in‐person (71.1%). A total of 784 CPs were tested, of whom 41.6% (326/784) were HIV positive. This included 157 persons newly diagnosed with HIV infection and 169 persons with a prior HIV diagnosis. Of the 326 HIV positive sexual partners, 43.2% had a CD4 count done and 24.5% were initiated on ART.

**Table 3:** Characteristics and HIV care outcomes of contact persons (CPs) notified

	CPs N = 1224	
	n	%
CPs HIV characteristics		
CPs notified		
Yes	1170	95.6
No	21	1.7
CP was dead	2	0.2
Missing	31	2.5
Method of notification		
Passive referral	535	43.7
Provider referral	542	44.3
Negotiated referral	62	5.1
Tested and diagnosed same time as index person	38	3.1
Missing	47	3.8
Phone or in‐person notification		
In‐person	870	71.1
Phone	293	23.9
Other	6	0.5
Missing	55	4.5
CPs tested for HIV		
Yes	784	64.1
No	235	19.2
Unknown	126	10.3
Missing	79	6.5

	n = 784	
HIV testing outcomes		
HIV status of CPs		
Newly diagnosed HIV positive	157	20.0
Previously diagnosed HIV positive	169	21.6
Tested HIV negative	458	58.4

	n = 326	
HIV Care outcomes for HIV positive CPs on follow‐up		
CD4 count done at diagnosing site		
Yes	141	43.2
No	26	8.0
Missing	159	48.8
CPs on anti‐retroviral therapy		
Yes	80	24.5
No	41	12.6
Missing	205	62.9

### 3.5 aPNS and social harms: sub‐set data from 2014 to 2015

Of the 1357 IPs enrolled, 267 (19.7%) reported a history of social harms before receiving aPNS from the sexual partners they mentioned (female IP: 24.2%, male IPs: 15.8%) with 7.4% of the IPs (101/1357) fearing for IPV from their sexual partners. Among those with a history of IPV, 43.5% (116/267) had experienced such harm in the three months before enrolment in the aPNS programme.

Adverse outcomes among IPs after receipt of aPNS were infrequent and many of them have not been directly related (Table 4). On follow‐up, 61 IPs reported subsequent adverse outcomes from their sexual partners (61/976, 6.3%), all of them involving partnership dissolution with 41.0% (25/61) of the remaining separate from their partners for at least two years (Table 4). Loss of financial support and physical IPV accounted for 1.5% and 1.1% of adverse outcomes among IPs after receipt of aPNS respectively. Overall, 11 (1.1%) of 976 persons for whom data was available reported physical IPV following their HIV diagnosis; and of these, three attributed the violence to aPNS provided by an HA. None required hospitalization.

**Table 4:** Adverse outcomes reported among index persons (IPs) at follow‐up

	IPs N = 976	
	n	%
Adverse outcomes reported at follow‐up		
Any adverse outcome (IPV, partnership dissolution, loss of financial support)	61/976	6.3
Partnership ended since testing HIV positive	61/976	6.3
Are you still separated from them?		
Yes	25/61	41.0
Duration of separation		
Less than two years	2/25	8.0
Two years	14/25	56.0
Three years	6/25	24.0
Greater than three years	3/25	12.0
No	10/61	16.4
Missing	26/61	42.6
Contact person (CP) left because IP tested HIV positive		
Yes	14/61	23.0
No	6/61	9.8
Don't know	17/61	27.9
Declined to answer	2/61	3.3
Missing	22/61	36.1
CP stopped financial support		
Yes	15/976	1.5
CP stopped financial support because of HIV positive result		
Yes	5/15	33.3
No	2/15	13.3
Don't know	5/15	33.3
Missing	3/15	20.0
No	18/976	1.8
Never received financial support from this CP	15/976	1.5
Declined to answer	1/976	0.1
Missing	12/976	1.2
Sustained physical IPV from CPs since testing HIV+		
Yes	11/976	1.1
Physical IPV from CP because of testing HIV positive	7/11	63.6
Physical IPV from CP because of HA notification	3/11	27.3
Reason missing	1/11	9.1
No	29/976	3.0
Don't know	15/976	1.5
Declined to answer	1/976	0.1
Missing	5/976	0.5

IPV, intimate partner violence; HA, health advisors.

## 4 Discussion

The programmatic outcomes of this large‐scale aPNS programme span nine years and demonstrate that implementation and scale‐up of aPNS were feasible in SSA with little social harm, despite limited funding. The primary benefits of aPNS are notification of individuals either at high risk of being HIV infected or those already HIV positive, facilitating confirmation of their HIV status, offering counselling, and referral services to care and treatment 4, 8, 15.

Most IPs were female, similar to a cluster randomized controlled trial in Kenya 10. Findings from a qualitative study conducted in Lesotho suggested that women have regular access to HTS through antenatal clinics and other maternal and child health‐related programmes. Also, men reported few opportunities to visit health clinics or HIV testing sites and commonly received information about HIV from mass media and friends. All participants unanimously characterized men as fearful and resistant to HIV testing 16. This perception matched reality in many SSA countries, where HIV testing has traditionally focused on women, and highlighted the value of aPNS for HIV case finding among men, a population often neglected in the healthcare system 17.

The CBCHS programme also integrated aPNS into other HIV and reproductive health services. Therefore, aPNS helped reduce the gender disparity in HIV case‐finding and referral to care and treatment services, with 67% of all CPs tested being men 17. Furthermore, studies suggested that male HIV‐testing was associated with increased acceptance and adherence among women attending PMTCT 18, 19 and was crucial to the success of PMTCT interventions designed to promote women's health 20, 21.

The mean CP/IP in the CBCHS aPNS programme ranged between 1.1 and 1.3, similar to results from a pilot aPNS programme in public urban clinics in Mozambique (1.4) 12, but lower than that found in Kenya (1.67) 10. This disparity could be due to the implementation of aPNS in real‐world conditions as a public health intervention in Cameroon and to a lesser extent in Mozambique, as compared to controlled research settings in Kenya. Also, the Cameroon aPNS programme started in faith‐based health facilities, which might have biased PLHIV, either in the choice of which facility to visit or in revealing information about their CPs. The range of sexual partners reported by the IPs is not available in the data analysed for this paper, but a previous report of the CBCHS aPNS programme reported a median of four lifetime sexual partners (range, 1 to 50) and one sexual partner (range, 1 to 50) in the last three years 13.

HIV prevalence among CPs tested in Cameroon (51.8%) was 14 times higher than the national prevalence (3.7%), suggesting that aPNS played a key role not only in identifying PLHIV and referring them to care and treatment but also in identifying HIV‐uninfected persons in HIV‐discordant partnerships with the objective of preventing HIV transmission to the HIV uninfected partner 22. Although the overall referral of HIV positive CPs to HIV care and treatment services was 66%, all of HIV positive CPs were referred to HIV care and treatment five years after the programme was initiated. During the early years of aPNS implementation, access to care and treatment in Cameroon was challenging due to the high costs, a limited number of HIV treatment centres and dedicated staff, stigma and the restrictive treatment initiation criteria based on CD4 count 24. With increased support from international funding partners, HIV care coverage has expanded nationally with more PLHIV were referred and initiated on ART 22, 23. The proportion of CPs who initiated ART remained in care and adhered to treatment were not systematically documented and therefore not reported in this evaluation.

Our findings related to social harms associated with aPNS are largely consistent with other studies. In Kenya, less than 3% of CPs who received aPNS experienced physical IPV, while few cases of partnership dissolution were notified in Malawi and Mozambique 10, 11, 12. These findings from various socio‐cultural settings provided evidence that aPNS, as implemented by trained providers, results in low levels of social harms. When our programme identified cases experiencing social harms, they were managed by experienced counsellors. All aPNS are voluntary; thus, when HAs elicit a history of social harms, they should proceed cautiously and IPs should be given an opportunity to decline aPNS.

The Cameroon Ministry of Public Health recognized CBCHS's commitment to increasing access to testing for those at higher HIV risk through aPNS and included it in national HIV control strategies and policies in 2014. This was a bottom‐up approach in the implementation of an effective HIV prevention strategy and its success was largely due to the unwavering determination of the CBCHS leadership and dedicated personnel. The CBCHS programme was influential in the early promotion and design of aPNS interventions and training of staff in other high HIV prevalence SSA countries including Kenya and Mozambique.

aPNS implementation in Cameroon had some limitations and challenges. Throughout the programme, limited funding greatly affected coverage and staff motivation, resulting in fewer CPs notified and tested, and poor follow‐up of IPs and HIV positive CPs. Obtaining accurate information from IPs was often difficult, as some gave incorrect information on their CPs, such as wrong phone numbers or wrong home address, and did not keep to their appointments. While there were concerns that aPNS may be associated with social harms and IPV, such outcomes occurred infrequently and the aPNS programme provided support and counselling to the affected individuals when such problems arose 13. A few CPs threatened HAs with violence, but none of the HAs experienced physical harm or litigation. Most of the monitoring and evaluation was based on aggregate data submitted monthly by participating facilities, which precluded disaggregation for more detailed analyses. The sub‐analysis of 2014 to 2015 data included information on the follow‐up of IPs. However, these analyses were not representative of the beneficiaries of the entire programme. The aPNS programme did not have sufficient resources to more actively follow‐up the CPs who were referred to HIV care and treatment, which likely would have allowed us to determine the proportion who received ART and to better identify social harms. aPNS provides the opportunity to link both IPs and HIV positive CPs to HIV care and treatment. ART is now universally available to all PLHIV in Cameroon. The NNTI numbers reported included all HIV positive CPs, both new diagnoses and people who may have been previously tested and not yet linked to ART.

## 5 Conclusions

Results presented from nine years (2007 to 2015) of the implementation of aPNS as a large‐scale public health programme shows that it has been feasible in Cameroon. aPNS helped identify many PLHIV who did not know their status, who were then counselled on HIV prevention strategies if HIV negative, or referred to HIV care if HIV positive. Social harms and IPV occurred infrequently after aPNS. The HAs provided support to people experiencing social harms to mitigate long‐term adverse consequences.

## Competing interests

The authors declare that they have no conflicts of interest.

## Authors’ contributions

PTM, EMK, EN, WN and TW supervised the implementation of the CBCHS programme and participated in designing this secondary analysis along with FTC, RS and MG. PTM, WN and TW reviewed the safety of programme participants. TW, EN, RS and MG were responsible for the statistical design of the analysis. EN, FTC, BW and WN analysed the data. FTC, EMK, WN and TW wrote the initial draft of the paper. All authors critically revised, read and approved the final manuscript.

## Supporting information


**Appendix S1.** Health advisors' pledge.
