# Supplementary material for: Assisted HIV partner notification services in resource‐limited settings: experiences and achievements from Cameroon
Source: J Int AIDS Soc. 2019 Jul 19;22(Suppl Suppl 3):e25310. doi: 10.1002/jia2.25310 (PMC6639669; doi:10.1002/jia2.25310)
Supplement: Supplementary file 1 — Appendix S1. Health advisors' pledge. [file JIA2-22-e25310-s001.pdf]

Cameroon Baptist Convention Health Services

Extended Forum of Care Program

HEALTH ADVISORS' PLEDGE.

- You have gone through trainings as health advisors.
- You understand the task that lies ahead of you.
- You will counsel, test and post counsel clients.
- You will interview and know all their sexual partners.
- This information shall be given you in confidence of your profession as a health advisor.

THE PLEDGE

Do you pledge to:

- Work with people who are living with HIV and those who are at the risk of being infected with HIV.
- Inform and to encourage them to stop the spread of the infection from person to person.
- Uphold their dignity and to keep private all information made available to you in the course of the discharge of your duties.
- Do everything possible to reduce social harms to the index persons and
- Do everything in your powers to minimize the spread of diseases in the community?

IF YOU DO, SAY I DO SO PLEDGE, GOD HELPING ME.

***This should be read out by the program mentor (DHS)**
